# Reduction of indole‐3‐acetic acid methyltransferase activity compensates for high‐temperature male sterility in Arabidopsis

**DOI:** 10.1111/pbi.12768

**Published:** 2017-07-27

**Authors:** Mohamad Abbas, Jorge Hernández‐García, Noel Blanco‐Touriñán, Norma Aliaga, Eugenio G. Minguet, David Alabadí, Miguel A. Blázquez

**Affiliations:** ^1^ Instituto de Biología Molecular y Celular de Plantas Consejo Superior de Investigaciones Científicas (CSIC)—Universidad Politécnica de Valencia Valencia Spain; ^2^ Present address: Plant and Crop Science Sutton Bonington Campus University of Nottingham Nottingham UK

**Keywords:** pollen, auxin, fertility

## Abstract

High temperature is a general stress factor that causes a decrease in crop yield. It has been shown that auxin application reduces the male sterility caused by exposure to higher temperatures. However, widespread application of a hormone with vast effects on plant physiology may be discouraged in many cases. Therefore, the generation of new plant varieties that locally enhance auxin in reproductive organs may represent an alternative strategy. We have explored the possibility of increasing indole‐3‐acetic acid (IAA) in ovaries by reducing IAA methyltransferase1 (IAMT1) activity in *Arabidopsis thaliana*. The *iamt1* mutant showed increased auxin signalling in funiculi, which correlated with a higher growth rate of wild‐type pollen in contact with mutant ovaries and premature ovule fertilization. While the production of seeds per fruit was similar in the wild type and the mutant at 20 °C, exposure to 29 °C caused a more severe decrease in fertility in the wild type than in the mutant. Loss of *IAMT1* activity was also associated with the production of more nodes after flowering and higher tolerance of the shoot apical meristem to higher temperatures. As a consequence, the productivity of the *iamt1* mutant under higher temperatures was more than double of that of the wild type, with almost no apparent trade‐off.

## Introduction

Global warming has been demonstrated to exert a negative effect on agriculture, for instance counteracting the possible benefits that an increase in CO_2_ might have in plant physiology (Peng *et al*., 2004). Among all the documented losses, rice grain production decreases 10% for every 1 °C rise in night temperature, and it has been estimated that every 1 °C increase over 15 °C reduces wheat production by 3%–4% (Wardlaw, 1994). Similar deleterious effects have been shown for maize and barley, for which each day that the plants are exposed to temperatures over 30 °C, yield is reduced in 1% (Lobell *et al*., 2011).

One of the most serious problems caused by higher temperatures with respect to cultivated plants is the decrease in male fertility, already reported for tomato, cotton, barley and rice (Lobell *et al*., 2011). The effect of high temperature stress on plant reproduction has been extensively studied (Hedhly *et al*., 2009; Jain *et al*., 2007; Kakani *et al*., 2005; Snider *et al*., 2011; Zinn *et al*., 2010) and include the inhibition of male and female gametophyte development; the inhibition of pollen germination; the limitation of pollen tube growth and the alteration of the development of tissues required to carry out reproductive processes (i.e. anther and pistil tissues).

The investigation of treatments and the search for crop varieties that allow an increased tolerance to high temperatures is a very active research field. Several compounds have been tested in the lab that improve pollen fertility with different degrees of success, such as ethylene, brassinosteroids and auxins (Firon *et al*., 2012; Singh and Shono, 2003). Given that all of these compounds are plant hormones that modulate multiple developmental processes, their application in the field might have secondary, undesired effects in yield, suggesting the need for new strategies that allow more selective hormone action without those secondary effects.

Among the plant hormones that induce temperature tolerance to pollen, auxin is the best studied. In Arabidopsis, higher‐order loss‐of‐function mutants of the auxin biosynthesis *YUCCA* genes show male sterility (Cheng *et al*., 2006), indicating that auxin is necessary for anther and pollen development. In agreement with this, their expression level is reduced by higher temperatures in Arabidopsis and also in barley (Sakata *et al*., 2010a), causing a decrease in auxin signalling in anthers (Sakata *et al*., 2010a,b). A key observation was that auxin application to flowers increased the reproductive tolerance to high temperatures (Oshino *et al*., 2011; Sakata *et al*., 2010a). Although the main effect of exogenous auxin may be restoring of pollen maturation under higher temperatures, the growth of pollen tubes is also enhanced *in vitro* by the presence of the hormone in the medium (Wu *et al*., 2008). Therefore, auxin accumulation in ovaries could also be a strategy for higher reproductive success under high temperature because it would extend the distance covered by the pollen tube inside the ovary (and thus the chances to fertilize an ovule) before it dies.

Control of auxin biosynthesis can be exerted at several key steps (Ljung, 2013). For example, auxin overproduction traits can be achieved by overexpression of *YUCCA* genes alone (Chen *et al*., 2014; Cheng *et al*., 2006; Hentrich *et al*., 2013; Kim *et al*., 2007; Won *et al*., 2011), and synergistic effects can be reached by overexpression of *YUCCA* genes together with *TAA* genes—which encode another auxin biosynthesis rate limiting enzyme (Mashiguchi *et al*., 2011). Alternatively, overexpression of *GH3* genes encoding enzymes that catalyze auxin conjugation to amino acids also alters auxin content and auxin‐dependent processes (Domingo *et al*., 2009; Park *et al*., 2007). However, the redundancy of *GH3* genes in higher plants (Kumar *et al*., 2012; Terol *et al*., 2006; Yang *et al*., 2015; Yuan *et al*., 2013) complicates a knockout strategy to generate varieties with higher auxin content.

Methylation of indole‐3‐acetic acid (IAA) has been shown to participate in auxin homeostasis. In Arabidopsis, this activity is encoded by the *IAMT1* gene, whose overexpression causes defects in auxin‐dependent processes, such as development of hyponastic leaves and reduced root gravitropism, attributable to a reduction of auxin (Qin *et al*., 2005). These observations are also in agreement with the hypothesis that Me‐IAA is an inactive form of IAA (Li *et al*., 2008). Therefore, we have investigated the potential use of reducing IAA methyltransferase activity as a method to enhance reproductive success in plants.

## Results

### IAMT1 controls auxin accumulation in ovaries

**Figure 1:**
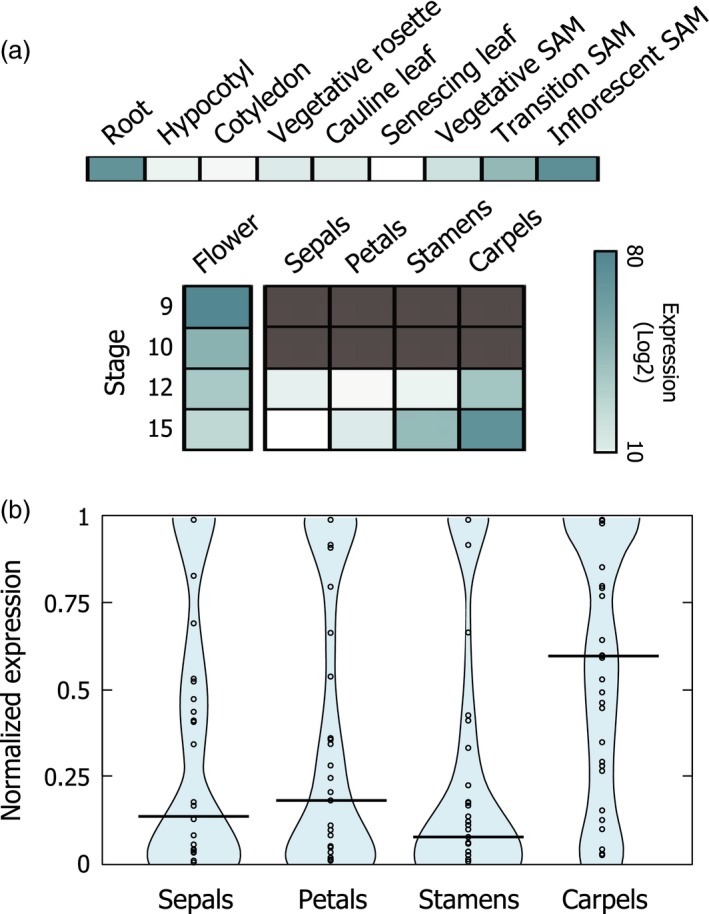
IAMT1 is expressed during pistil development. (a) Expression of *IAMT1* in flower tissues according to transcriptomic data from AtGenExpress Database. Dark grey color indicates no data available. (b) Beanplot view normalized expression values of the first 30 *IAMT1* co‐expressed genes in floral tissues.

The *IAMT1* gene is widely expressed in all Arabidopsis organs (Qin *et al*., 2005). Examination of public transcriptomic databases indicates that flowers display high levels of the *IAMT1* transcript, particularly in reproductive organs, such as ovaries and stamens (Figure 1a). Similarly, the expression of the top 30 genes with highest co‐expression values with respect to *IAMT1* are also high in ovaries, compared with other floral tissues (Figure 1b; Table S1). This observation suggests that auxin methylation might participate in the regulation of auxin accumulation in those organs. In fact, overexpression of *IAMT1* caused a decrease in auxin signalling determined by the expression level of the synthetic auxin reporter *DR5::GUS* (Qin *et al*., 2005), and silencing of *IAMT1* through RNA interference produced defects in fruit formation (Qin *et al*., 2005). However, it is not possible to rule out that these defects are the consequence of the unspecific reduction in the expression of other methyltransferases. Therefore, we examined auxin accumulation in the T‐DNA insertion mutant *iamt1‐1* (see Experimental procedures). Indeed, we could detect higher *DR5::GUS* expression levels in pre‐anthesis ovaries of the *iamt1* mutant than in wild‐type ovaries (Figure S1a). This effect was visible in siliques, more specifically in the funiculi (Figure S1b), where high expression level of *IAMT1* has been reported (Qin *et al*., 2005). Nevertheless, this enhanced response to auxin did not cause parthenocarpic fruit development (Figure S1c), as could have been expected from the effects of exogenous auxin applications (Alabadi *et al*., 2009; Dorcey *et al*., 2009).

### Attenuation of IAMT1 activity promotes fertilization

**Figure 2:**
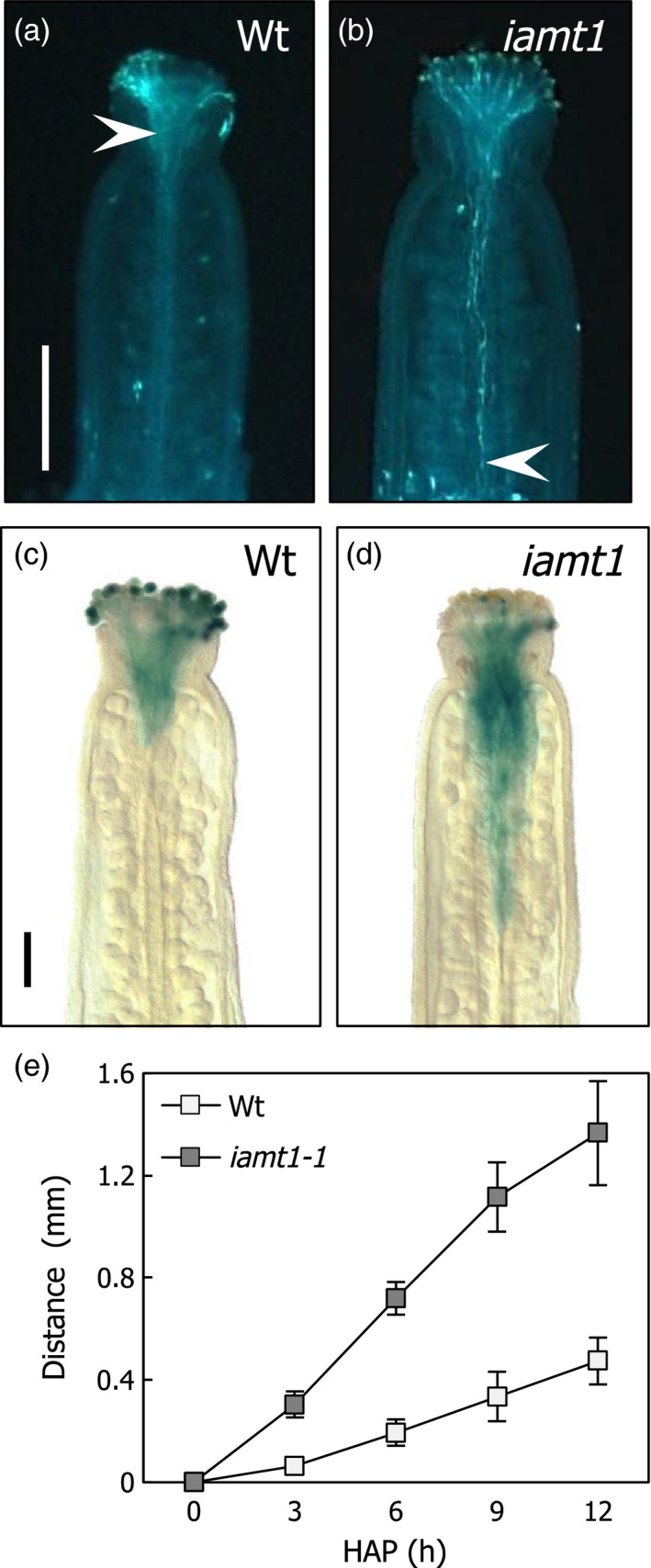
Faster pollen tube growth in *iamt1* ovaries. Progress of pollen tubes was visualized with callose (a, b) and *GA20ox1::GUS* (c, d) staining of wild‐type and *iamt1* ovaries 6 h after manual application of wild‐type pollen. (e) Pollen tube growth rate determined after pollination of wild‐type and *iamt1* mutant ovaries with wild‐type pollen. Arrowheads in (a) and (b) mark the point reached by pollen tubes. Scale bars in (a) and (c) are 0.4 and 0.2 mm, respectively.

To test if loss of IAMT1 function could lead to changes in pollen tube growth equivalent to the acceleration achieved by auxin treatments (Wu *et al*., 2008), we determined the length of pollen tubes in manually pollinated wild‐type and *iamt1* mutant ovaries of flowers in pre‐anthesis. The trajectory of pollen tubes in the ovaries was visualized using callose staining, or taking advantage of the high expression level of *GA20ox1* in mature pollen and using pollen from a *GA20ox1::GUS* line (Plackett *et al*., 2012). Figure 2 shows how pollen tubes covered a longer distance 6 h after hand‐pollination in the mutant ovaries. Indeed, the speed of pollen tubes was more than double in *iamt1* mutant than in wild‐type ovaries during the first 12 h after pollination (Figure 2e).

**Figure 3:**
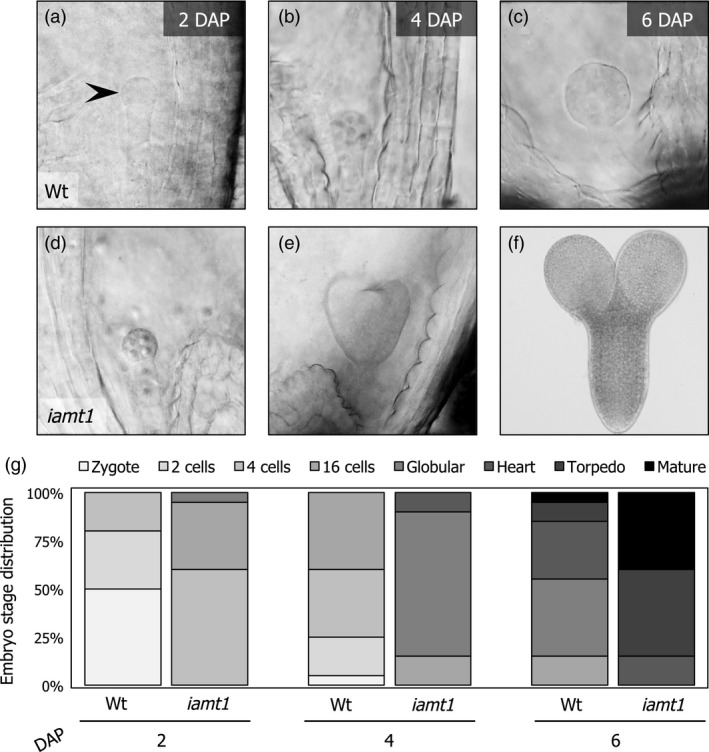
Fertilization occurs earlier in *iamt1* mutant ovaries. Pictures of representative embryo development stages in wild‐type (a–c) and *iamt1* ovaries (d–f) 2, 4 and 6 days after manual application of the corresponding pollen. (g) Quantification of embryo stages as a function of time after manual pollination (at least 250 embryos were analysed per time point).

More importantly, the faster pollen tube growth in the *iamt1* ovaries seemed to have biologically relevant consequences because, on average, embryos dissected from mutant ovaries at different time points after manual pollination were in a later stage of development compared to those from wild‐type ovaries (Figure 3). This is in agreement with an earlier fecundation of the ovules in the *iamt1* mutant.

Therefore, loss of IAMT1 activity seems to increase the capacity of ovule fecundation, which might be particularly beneficial in situations in which fecundation becomes compromised.

### Loss of IAMT1 activity enhances thermotolerance

**Figure 4:**
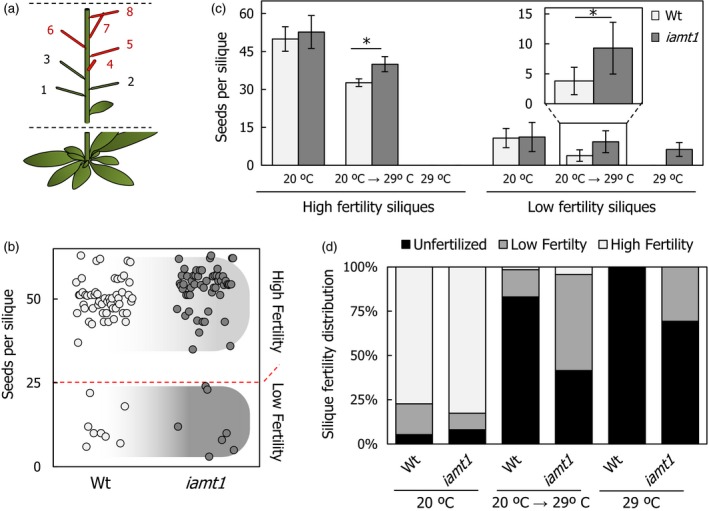
Increased seed production by *iamt1* fruits at higher temperature. (a) Representation of siliques measured in our approach (numbers 4–8). (b) Silique fertility phenotype discrimination threshold set up based in a seeds‐per‐silique dot‐blot of wild‐type and *iamt1* plants grown at 20 °C. (c) Seeds per silique measurements of wild‐type and *iamt1* plants grown under different temperature regimes. (d) Distribution of silique fertility phenotypes from plants grown at different temperature conditions. ‘Unfertilized’ stands for siliques with no mature seeds. Asterisk indicates *P* < 0.01 (Student's t‐test).

Considering that higher temperatures not only interfere with pollen development (Kim *et al*., 2001), but also quickly reduce pollen tube growth (Kakani *et al*., 2002), we hypothesized that faster pollen tube growth rates in *iamt1‐1* could result in higher reproductive capacity of the mutant under temperature stress. To test this possibility, we compared the production of seeds in individual fruits of Arabidopsis plants grown in three conditions: at constant 20 °C, at 29 °C from germination or transferred from 20 to 29 °C right after bolting. In all cases, the fruits examined were the siliques in nodes 4–8 (Figure 4a). Two classes of fruits could be distinguished, irrespective of the genotype and condition: low‐ and high‐fertility siliques, if they contained below or over 25 seeds, respectively (Figure 4b). No significant difference was found between the wild type and the *iamt1‐1* mutant at 20 °C and although exposure to 29 °C dramatically reduced the fertility of wild type and mutant plants, both classes of siliques consistently contained a higher number of seeds in *iamt1‐1* than in the wild type under those conditions (Figure 4c,d). This effect is in agreement with the observed increase in pollen tube growth in *iamt1‐1* ovaries and not in pollen, considering that pollen from *iamt1‐1* mutant flowers at 29 °C was as little viable as that from wild‐type plants (Figure S2).

**Figure 5:**
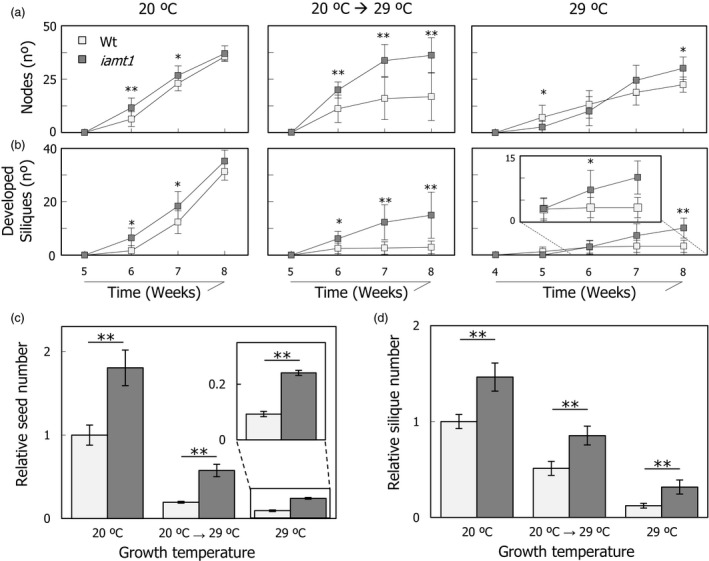
Total productivity of *iamt1* plants. Node (a) and silique (b) formation rates. Relative variation in total number of seeds (c) and siliques (d) produced by wild‐type and *iamt1* plants grown at different conditions compared to 20 °C grown wild‐type production. To calculate relative values, total number of seeds (or siliques) was counted from a total of nine plants per genotype and condition as explained in Experimental procedures, the mean values were obtained in each case, and then all values were referred to that of wild type at 20 °C. One and two asterisks indicate *P* < 0.01 and *P* < 0.001, respectively (Student's *t*‐test).

The mutant also displayed a differential behaviour under temperature stress when other unrelated traits were examined. For instance, the mutant produced more nodes than the wild type after exposure to 29 °C (Figure 5a), a higher number of fully developed siliques (Figure 5b) and an enhanced tolerance of the shoot apical meristem to higher temperature (Figure S3). As a result, a general increase in productivity was observed in *iamt1‐1* mutant plants at all temperatures, measured as the total number of fruits and seeds per plant (Figure 5c,d).

**Figure 6:**
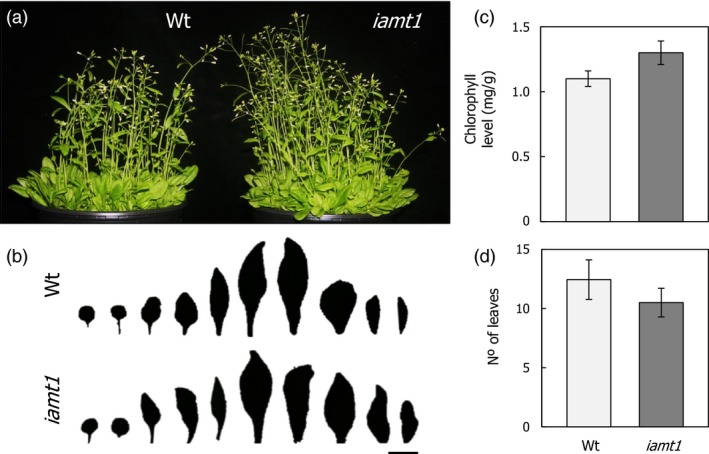
Phenotypic characterization of the *iamt1* mutant. (a) Picture of the plants. (b) Size of leaves. (c) Chlorophyll levels. (d) Flowering time in long days. Bar length, 1 cm.

The phenotype of the *iamt1‐1* mutant under higher temperatures suggests that *IAMT1* could be considered a potentially useful biotechnological target to increase reproductive capacity in plants under stress. To confirm the suitability of such an approach, we examined in detail possible deleterious effects caused by loss of IAMT1 function. All developmental traits analysed at normal growth temperature were neutral in the *iamt1‐1* mutant, such as no decrease in plant size (Figure 6a,b), similar chlorophyll level (Figure 6c), and a slight acceleration of flowering time (Figure 6d; Figure S4). More importantly, the seeds produced by the *iamt1‐1* mutant at 29 °C had a similar size and germination ability as those of the wild type (Figure S5), ruling out a possible penalty caused by loss of IAMT1 function.

## Discussion

Manipulation of auxin metabolism or auxin signalling has already been successfully explored as a biotechnology tool with different purposes. For instance, introduction of a bacterial *iaaM* gene in eggplant under the control of an ovule‐specific promoter caused the formation of marketable seedless fruits which could be produced even under environmental conditions that normally do not allow fruit set (Rotino *et al*., 1997), and this strategy is also applicable to other species within the Rosaceae (Mezzetti *et al*., 2004). The involvement of auxin in cotton fibre formation has also led to the improvement of cotton fibre yield and quality by targeted expression of *iaaM* to the epidermis of cotton ovules (Zhang *et al*., 2011). And the recent introduction of the *iaaM* gene in potato, driven by the tuber‐specific promoter of a patatin gene, has been reported to result in an increase in tuber productivity even under unfavourable conditions (Kolachevskaya *et al*., 2015). Moreover, there are indications that biotechnological modification of the auxin pathway can also be extended to stress resistance, since ectopic overexpression of the Arabidopsis *YUCCA6* gene in potato, despite showing agronomically undesirable traits, enhances drought tolerance (Kim *et al*., 2013).

The results shown here suggest that down‐regulation of IAMT1 function is a novel potential strategy to generate crop varieties with reduced loss of productivity under high temperature stress. Compared to previous approaches to alter auxin‐related traits, the manipulation of IAA methyltransferase activity presents several advantages: (i) it is based on a loss‐of‐function approach, rather than overexpression, therefore allowing the search for knockdown or knockout alleles in crops using TILLING (Till *et al*., 2003) or CRISPR‐Cas9 (Jiang *et al*., 2013); (ii) it takes advantage of the fairly specific expression pattern of *IAMT1*, avoiding the need to use more sophisticated cell type‐specific approaches and preventing the secondary effects of unrestricted auxin application and (iii) the conservation of the IAMT1 protein sequence in monocots (Zhao *et al*., 2008; Zubieta *et al*., 2003) suggests the possible use of this strategy in the majority of relevant crops, including grasses.

Although our examination of developmental traits in the Arabidopsis *iamt1* mutant showed no significant differences with respect to the wild type, this analysis did not rule out the possibilities that the loss of *IAMT1* affects (positively or negatively) other traits associated with biotic or abiotic stress resistance. This is likely, given that the *IAMT1* gene is highly expressed in roots (Figure 1) (Qin *et al*., 2005), and auxin has been implicated in mediating the adaptation of root architecture to environmental stress (Kazan, 2013).

How *IAMT1* down‐regulation promotes pollen tube growth is still unknown. The observation that altering local auxin levels in the ovary alone (e.g. the funiculi) favours polar expansion of pollen tubes along the transmitting tract indicates that auxin either acts in the ovary itself, or enters pollen cells. There are observations that support each of the two possibilities. On one hand, it has been reported that auxin participates in the differentiation of ovary tissues, including funiculi and transmitting tract, through the HECATE transcription factors (Gremski *et al*., 2007). And on the other hand, intracellular auxin has been shown to drive polar growth of pollen tubes through the control of vesicle trafficking (Dal Bosco *et al*., 2012; Ding *et al*., 2012). In any case, the involvement of IAMT1 in the control of pollen tube growth identifies not only a biotechnological aid, but also a tool to dissect the molecular events following pollination.

## Experimental procedures

### Plant material and growth

*Arabidopsis thaliana* ecotype Col‐0 was used as wild type. Transgenic lines used were already published: *DR5::GUS* (Sabatini *et al*., 1999), *GA20ox2::GUS* (Plackett *et al*., 2012). The *DR5::GUS* transgene was introgressed in the *iamt1‐1* mutant by crossing. The *iamt1‐1* corresponds to the T‐DNA insertion line SALK_072125 (Alonso *et al*., 2003). This line was genotyped with *IAMT1*‐specific oligonucleotides and with an oligonucleotide specific for the T‐DNA left border (Table S2). Transgene presence in progeny was determined by the corresponding antibiotic resistance and by genotyping (Figure S6).

For adult plant growth, seeds were stratified in water for 3 days at 4 °C, sown on pots containing soil mix (1 : 1 : 1 perlite, vermiculite and peat) and grown in Percival growth chambers under long day conditions (16 h of light and 8 h of darkness). Different temperature conditions were used depending on the experiments: constant 20 °C was used unless specified, while constant 29 °C from germination, or vegetative growth at 20 °C from germination and transference to 29 °C right after bolting were used in temperature stress assays. For *in vitro* growth, seeds were surface sterilized and sown on ½ MS plates with 1% (w/v) sucrose, 8 g/L agar, pH 5.8. Seeds were stratified for 3 days at 4 °C, and grown in growth chambers under long day conditions.

### Silique and seed production analyses

For parthenocarpy assays, at least 15 pre‐anthesis flowers (stage 12) were emasculated in two replicates. Growth was followed until siliques were developed in manually pollinated pistils used as control.

Silique and node formation rates were followed on main stems of at least 15 individual soil growing plants per genotype and temperature during 4–5 weeks after bolting and to global proliferative arrest or final senescence of the plant. Buds and flowers until stage 16–17 (or obvious silique development) were counted as nodes, and siliques from stage 16 as developing siliques.

For seed production in individual siliques, 4th to 8th siliques of main stems were gathered individually after ripening from at least 15 individual plants. Silique fertility was divided in three different phenotypic categories according to seed production in standard conditions. Siliques producing more or <25 seeds were defined as high‐fertility or low‐fertility siliques, respectively. When no seeds were produced, these nodes or partially developed siliques were only accounted for fertility distribution. Collected seeds from individual ripening siliques were counted, measured as previously described (Herridge *et al*., 2011), and stored.

For total seed and silique production, three plants grown in soil at the different temperature conditions were randomly chosen, and siliques and seeds were counted manually under a stereoscope. The experiment was repeated three times, and the corresponding means and standard errors were calculated.

For germination assays, seeds harvested at the same time were stored for 3 weeks at 4 °C, and germinated *in vitro*. Radicle emergence was counted as germination (Bewley *et al*., 2013). The area of the seed surface was measured using ImageJ software (University of Wisconsin, Madison, Wisconsin) on photographs of the seeds taken under the stereoscope. Values were analysed with the two‐tailed Student′s *t*‐test.

### Expression and co‐expression analyses

*IAMT1* expression levels in different developmental stages were obtained from AtGenExpress database (The Arabidopsis Information Resource, The Ohio State University, OH). Graphic heatmap representations were constructed using the Matrix2png website (Pavlidis and Noble, 2003). The first 30 genes co‐expressed with *IAMT1* were retrieved from the ATTED‐II database (Aoki *et al*., 2016) and their expression values in flower organs (stage 12) obtained from AtGenExpress (Table S1). Expression values in flower organs were normalized individually in all the genes and analysed using BoxPlotR (Spitzer *et al*., 2014).

### Pollen tube growth assays

Pre‐anthesis flowers (stage 12) were emasculated and pistils manually pollinated either with wild‐type or *GA20ox1:GUS* line pollen. Pistils were collected 6 h after manual pollination unless specified and subjected either to callose or GUS staining. Callose staining was performed with aniline blue as described (Jiang *et al*., 2005) and observed under UV irradiation and a DAPI filter set in a Nikon Eclipse E600 microscope (Nikon, Barcelona, Spain). GUS staining was performed as previously described (Jefferson *et al*., 1987) using 50 mm phosphate buffer (pH 7.0) containing 0.1% (v/v) Triton X‐100, 1 mm potassium ferrocyanide, 1 mm potassium ferricyanide and 2 mm X‐Gluc. Staining was examined after overnight incubation at 37 °C in a Nikon Eclipse E600 microscope. Pollen tube measurements were done on callose stained pictures with imageJ software with at least 10–15 individually pollinated pistils per genotype in two biological replicates.

### Chlorophyll measurements

For chlorophyll extraction, leaves from adult plants were used. Leaves were frozen and crushed with a mortar and pestle in liquid nitrogen. Hundred milligrams of tissue was extracted in 2 mL of ice cold acetone (80%) for an hour in darkness at 4 °C. The chlorophyll content was calculated using spectrophotometric absorbance (*A*) at wavelengths of 645 and 663 nm and 80% acetone as a control, and shown as milligrams of chlorophyll per gram of fresh tissue as follows: Chlorophyll *a* (mg/g) = 12.7 × *A*_663_ − 2.69 × *A*_645_; chlorophyll *b* (mg/g) = 22.9 × *A*_645_ − 4.86 × *A*_663_; and chlorophyll *a* + *b* (mg/g) = 8.02 × *A*_663_ + 20.20 × *A*_645_ as described before (Abbas *et al*., 2015).

### Embryo extraction, imaging and quantification

For embryo extraction, ten green fruits were chosen 2, 4, and 6 days after manual pollination. The replum was dissected using a needle under the stereoscope; ovules were collected, placed directly in chloral hydrate (Sigma‐Aldrich Quimica SL, Madrid, Spain), gently crushed with a micro‐pestle, and left 2 h in darkness for clearing. Images were taken at the appropriate magnification in a Nikon Eclipse E600 microscope. Quantification was done manually.

## Supporting information


**Figure S1** (a) Increased levels of *DR5::GUS* expression in preanthesis ovaries. Bar length = 100 μm. (b) Localized increase in auxin activity in the funiculi of *iamt1* mutant fruits, shown by *DR5::GUS* staining. Bar length = 500 μm (full fruit), 200 μm (close up). (c) The *iamt1* mutant is not parthenocarpic. Bar length = 2 mm. P, pollinated; UP, unpollinated.
**Figure S2** Loss of IAMT1 function in pollen does not protect from higher temperature stress.
** S3** Shoot apical meristems (SAM) from *iamt1* mutants are partially resistant to temperature stress conditions.
**Figure S4** Flowering time measured in days of wild‐type and *iamt1* mutant plants grown under short and long days.
**Figure S5** Seeds produced at 29 °C are normal.
**Figure S6** Scheme of the *IAMT1* locus indicating the position of the T‐DNA insertion of *iamt1‐1* and the genotyping result.
**Table S1** Top 30 genes with highest co‐expression levels with respect to *IAMT1*.
**Table S2** DNA sequence of oligonucleotide primers used for genotyping.
