# Supplementary material for: Reduction of indole‐3‐acetic acid methyltransferase activity compensates for high‐temperature male sterility in Arabidopsis
Source: Plant Biotechnol J. 2017 Jul 27;16(1):272–9. doi: 10.1111/pbi.12768 (PMC5785359; doi:10.1111/pbi.12768)
Supplement: Supplementary file 1 — Figure S1 (a) Increased levels of DR5::GUS expression in preanthesis ovaries. Bar length = 100 μm. (b) Localized increase in auxin activity in the funiculi of iamt1 mutant fruits, shown by DR5::GUS staining. Bar length = 500 μm (full fruit), 200 μm (close up). (c) The iamt1 mutant is not parthenocarpic. Bar length = 2 mm. P, pollinated; UP, unpollinated. Figure S2 Loss of IAMT1 function in pollen does not protect from higher temperature stress. Figure S3 Shoot apical meristems (SAM) from iamt1 mutants are partially resistant to temperature stress conditions. Figure S4 Flowering time measured in days of wild‐type and iamt1 mutant plants grown under short and long days. Figure S5 Seeds produced at 29 °C are normal. Figure S6 Scheme of the IAMT1 locus indicating the position of the T‐DNA insertion of iamt1‐1 and the genotyping result. Table S1 Top 30 genes with highest co‐expression levels with respect to IAMT1. Table S2 DNA sequence of oligonucleotide primers used for genotyping. [file PBI-16-272-s001.pdf]

Table S1. First 30 genes with highest co-expression values with respect to IAMT1

	Locus	Alias	Function	Supportability	Ath-m
0	**At5g55250**	IAMT1	IAA carboxylmethyltransferase 1	1	0
1	**At5g24910**	ELA1	cytochrome P450, family 714, subfamily A, polypeptide 1	0	13
2	**At3g22540**	DUF1677	Protein of unknown function (DUF1677)	2	15,5
3	**At4g22460**	inhibitor	Bifunctional inhibitor/lipid-transfer protein/seed storage 2S albumin superfamily protein	2	17,3
4	**At4g33790**	G7/CER6	Jojoba acyl CoA reductase-related male sterility protein	1	22,3
5	**At5g24410**	PGL4	6-phosphogluconolactonase 4	2	24,4
6	**At4g22110**	dehydrogenase	GroES-like zinc-binding dehydrogenase family protein	2	27,1
7	**At1g12550**	HPR3	D-isomer specific 2-hydroxyacid dehydrogenase family protein	0	34,6
8	**At2g03370**	transferase	Glycosyltransferase family 61 protein	1	39,7
9	**At3g14850**	TBL41	TRICHOME BIREFRINGENCE-LIKE 41	1	40,7
10	**At5g15180**	Peroxidase	Peroxidase superfamily protein	2	51,6
11	**At3g62610**	MYB11/PFG2	myb domain protein 11	1	54
12	**At4g25830**	UPF0497	Uncharacterised protein family (UPF0497)	2	55,4
13	**At5g07990**	TT7/CYP75B1	Cytochrome P450 superfamily protein	3	60,9
14	**At5g51810**	GA20OX2	gibberellin 20 oxidase 2	0	62,2
15	**At2g43050**	PMEPCRD	Plant invertase/pectin methylesterase inhibitor superfamily	1	64,7
16	**At4g34500**	kinase	Protein kinase superfamily protein	1	66,1
17	**At5g39220**	alpha/beta-Hydrolases	alpha/beta-Hydrolases superfamily protein	2	68
18	**At4g15480**	UGT84A1	UDP-Glycosyltransferase superfamily protein	2	69,3
19	**At3g28420**	Putative membrane lipoprotein	Putative membrane lipoprotein	2	69,9
20	**At3g18170**	transferase	Glycosyltransferase family 61 protein	2	71,8
21	**At1g25510**	protease	Eukaryotic aspartyl protease family protein	2	74,8
22	**At1g49800**	PIPL1	PAMP-INDUCED SECRETED PEPTIDE (PIP) LIKE (PIPL)	1	76,5
23	**At4g01140**	DUF1191	Protein of unknown function (DUF1191)	2	77
24	**At2g22620**	lyase	Rhamnogalacturonate lyase family protein	2	82,2
25	**At5g01200**	Duplicated homeodomain-like	Duplicated homeodomain-like superfamily protein	0	82,5
26	**At1g51170**	UCN	Protein kinase superfamily protein	1	83,8
27	**At1g11590**	inhibitor	Plant invertase/pectin methylesterase inhibitor superfamily	1	86,5
28	**At1g66460**	kinase	Protein kinase superfamily protein	0	87,4
29	**At5g26660**	MYB86	myb domain protein 86	1	107,1
30	**At5g56300**	GAMT2	gibberellic acid methyltransferase 2	1	110,6

Table S2: DNA sequence of oligonucleotide primers used for genotyping.

**Name**	**AGI number**	**Forward (5' -3')**	**Reverse (5'-3')**
SALK_072125	AT5G55250	TTAAAGAGAGAAGGAGAGATCCATAGAGA	CATGGCCCCACCTCTCTTAA
LBb1.3		GTATAGTTCATCCATGCCATGTG	
GUS		CAACGTCTGCTATCAGCGCGAAGT	TATCCGGTTCGTTGGCAATACTCC

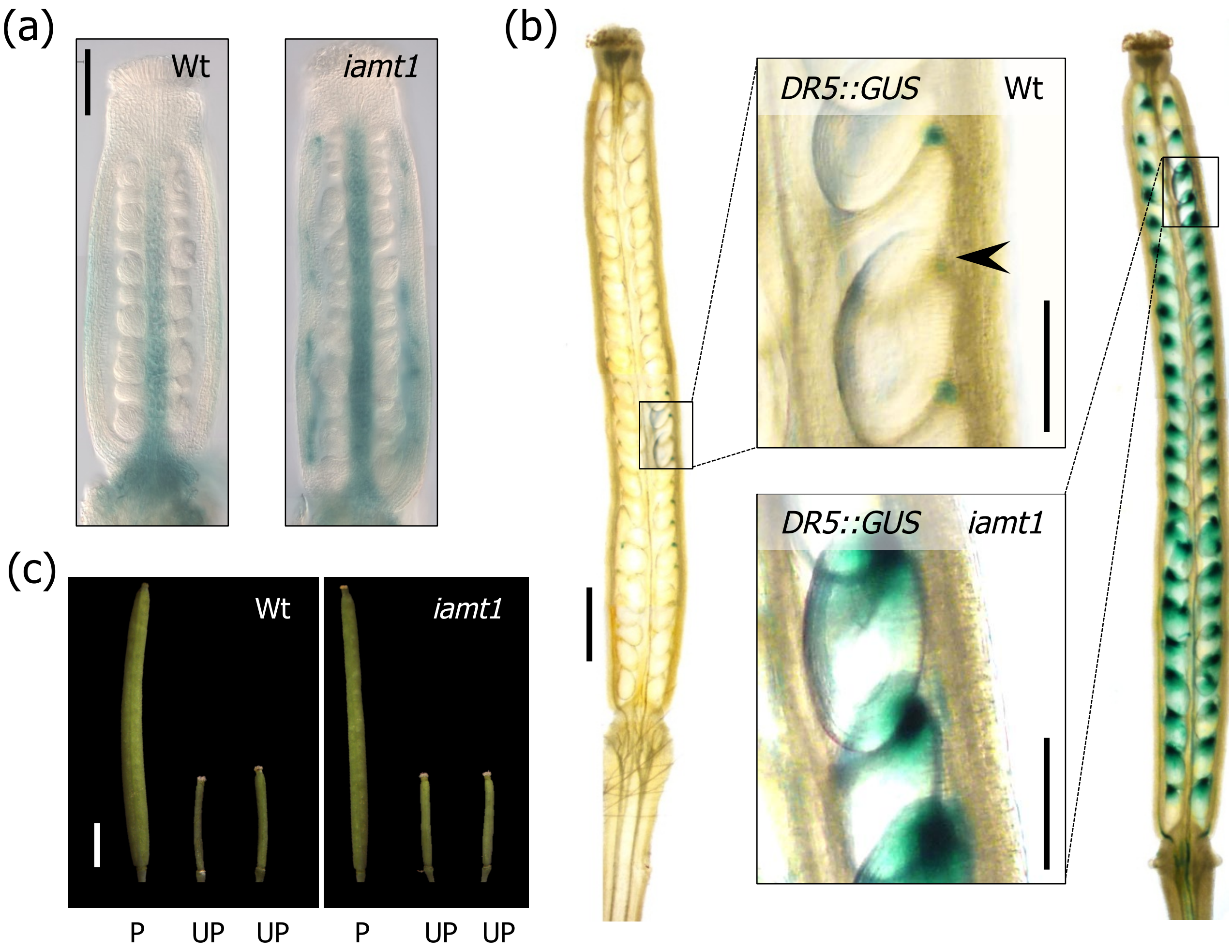

Figure S1. (a) Increased levels of *DR5::GUS* expression in preanthesis ovaries. Bar length = 100 µm. (b) Localized increase in auxin activity in the funiculi of *iamt1* mutant fruits, shown by *DR5::GUS* staining. Bar length = 500 µm (full fruit), 200 µm (close up). (c) The *iamt1* mutant is not parthenocarpic. Bar length = 2mm. P, pollinated; UP, unpollinated.

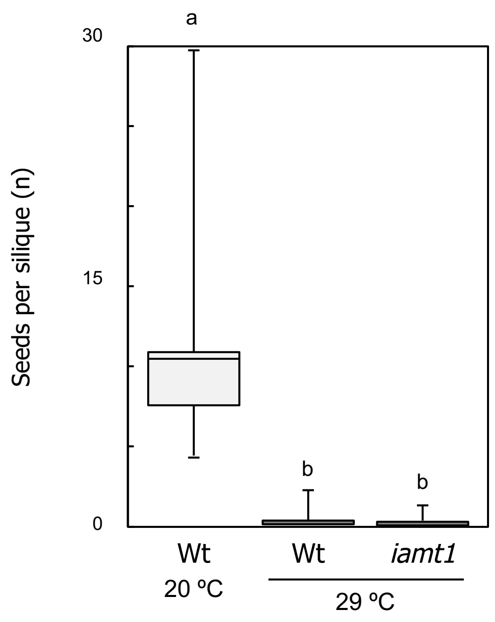

Figure S2. Loss of IAMT1 function in pollen does not protect from higher temperature stress. Wild-type and *iamt1* mutant plants were transferred from 20 to 29ºC for 7 days after bolting, and pollen from newly produced flowers was used to manually pollinate wild-type plants at 20ºC. As a control, wild-type pollen at 20ºC was also used. At least 12 flowers per genotype were examined. Letters indicate significant differences between groups, $p < 0.01$ (One way ANOVA, Tukey HSD Post Hoc test).

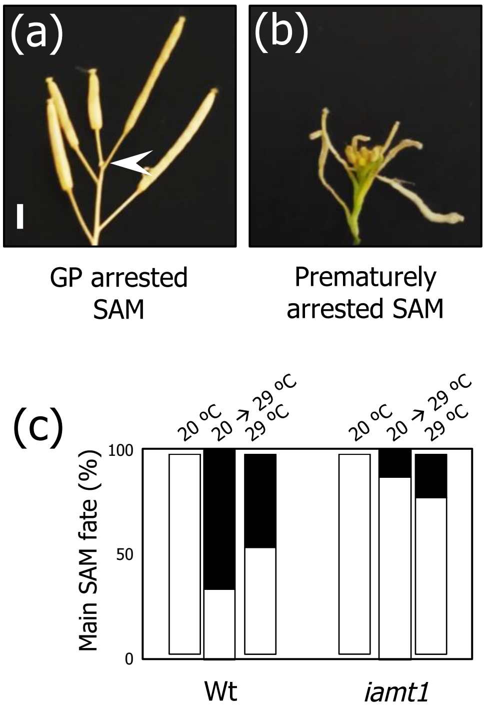

Figure S3. Shoot apical meristems (SAM) from *iamt1* mutants are partially resistant to temperature stress conditions. Illustration of SAM phenotypes of wild-type plants grown under standard (a) or higher (b) temperatures. (c) Quantification of SAM fate under different temperature conditions. White and black colours represent normal and prematurely arrested, respectively. "GP" stands for "Global Proliferative".

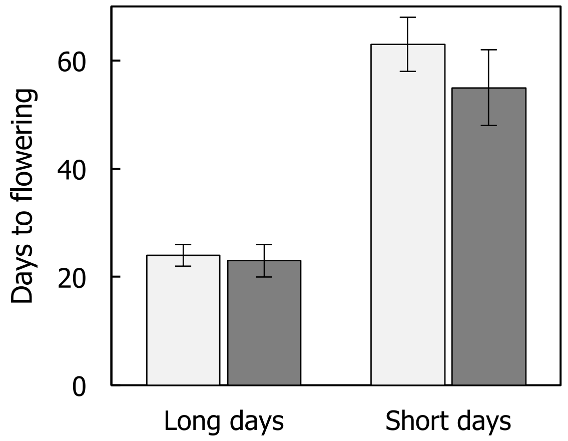

Figure S4. Flowering time measured in days of wild-type and *iamt1* mutant plants grown under short and long days.

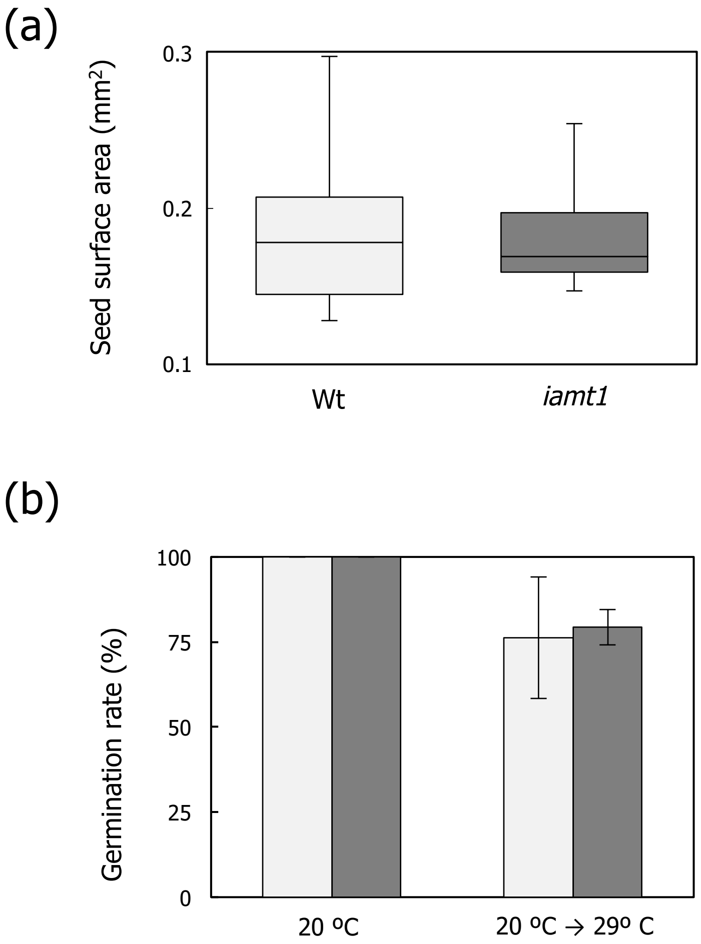

Figure S5. Seeds produced at 29ºC are normal. (a) Surface area of the seeds produced at 29ºC by wild-type and mutant plants (n=100). (b) Germination rate of seeds collected from wild-type and *iamt1* mutant pants growing at 20ºC or transferred to 29ºC. Germination was assessed 5 days after sowing.

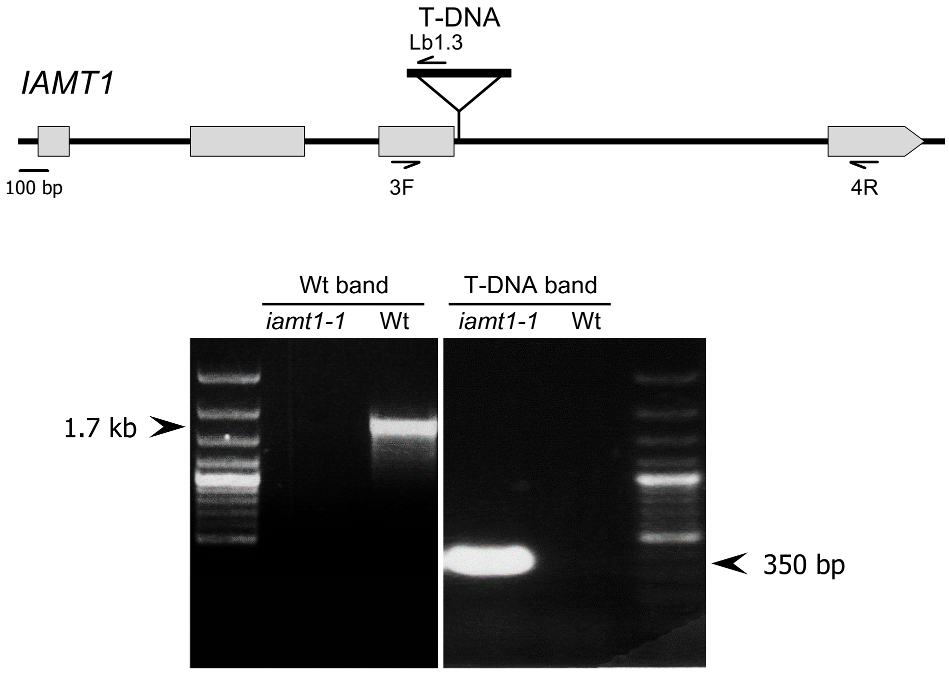

Figure S6. Scheme of the *IAMT1* locus indicating the position of the T-DNA insertion of *iamt1-1* and the genotyping result.
