# Supplementary material for: Reduction of indole‐3‐acetic acid methyltransferase activity compensates for high‐temperature male sterility in Arabidopsis
Source: Plant Biotechnol J. 2017 Jul 27;16(1):272–9. doi: 10.1111/pbi.12768 (PMC5785359; doi:10.1111/pbi.12768)
Supplement: Supplementary file 2 — Supplementary File [file PBI-16-272-s002.docx]

**Supplementary material**

**Figure S1.** (a) Increased levels of *DR5::GUS* expression in preanthesis ovaries. Bar length = 100 μm. (b) Localized increase in auxin activity in the funiculi of *iamt1* mutant fruits, shown by *DR5::GUS* staining. Bar length = 500 μm (full fruit), 200 μm (close up). (c) The *iamt1* mutant is not parthenocarpic. Bar length = 2mm. P, pollinated; UP, unpollinated.

**Figure S2.** Loss of IAMT1 function in pollen does not protect from higher temperature stress. Wild-type and *iamt1* mutant plants were transferred from 20 to 29ºC for 7 days after bolting, and pollen from newly produced flowers was used to manually pollinate wild-type plants at 20ºC. As a control, wild-type pollen at 20ºC was also used. At least 12 flowers per genotype were examined. Letters indicate significant differences between groups, p < 0.01 (One way ANOVA, Tukey HSD Post Hoc test).

**Figure S3.** Shoot apical meristems (SAM) from *iamt1* mutants are partially resistant to temperature stress conditions. Illustration of SAM phenotypes of wild-type plants grown under standard (a) or higher (b) temperatures. (c) Quantification of SAM fate under different temperature conditions. White and black colours represent normal and prematurely arrested, respectively. "GP" stands for "Global Proliferative".

**Figure S4.** Flowering time measured in days of wild-type and *iamt1* mutant plants grown under short and long days.

**Figure S5.** Seeds produced at 29ºC are normal. (a) Surface area of the seeds produced at 29C by wild-type and mutant plants (n=100). (b) Germination percentage of seeds collected from wild-type and *iamt1* mutant plants growing at 20ºC or transferred to 29ºC. Germination was assessed 5 days after sowing.

**Figure S6.** Scheme of the *IAMT1* locus indicating the position of the T-DNA insertion of *iamt1-1* and the genotyping result.

**Table S1.** Top 30 genes with highest co-expression levels with respect to *IAMT1*.

**Table S2.** DNA sequence of oligonucleotide primers used for genotyping.
